# Profile of Phenolic Compounds and Antioxidant Activity of Celery (*Apium graveolens*) Juices Obtained from Pulp after α-Amylase Treatment from *Aspergillus oryzae*

**DOI:** 10.3390/molecules29071438

**Published:** 2024-03-23

**Authors:** Natalia Szarek, Grażyna Jaworska, Paweł Hanus

**Affiliations:** 1 Doctoral School of the University of Rzeszow, University of Rzeszow, Rejtana 16C Street, 35-959 Rzeszow, Poland; 2 Department of Food Technology and Human Nutrition, Institute of Food Technology and Nutrition, College of Natural Sciences, University of Rzeszow, Zelwerowicza 4 Street, 35-601 Rzeszow, Polandphanus@ur.edu.pl (P.H.)

**Keywords:** enzymatic maceration, pressing yield, phenolic compounds, sugar content

## Abstract

The purpose of this study was to determine the content of certain phenolic compounds, antioxidant activity, pressing efficiency, extract content, and sugars in celeriac juices obtained from the pulp after α-amylase treatment from *Aspergillus oryzae*. The test material consisted of peeled and unpeeled celery pulp kept at a temperature of 25 °C with and without the enzyme for a period of 30 and 60 min. The juices obtained from them were analyzed for the content of selected phenolic acids and flavonoids using the UPLC-PDA-ESI-MS/MS method, for antioxidant activity measured using the ABTS˙^+^ and DPPH˙ method, and for the total polyphenol content using the F-C method. Additionally, the juice pressing efficiency, the extract content using the refractometer method, and the sugar content using the HPLC method were checked. Significantly higher antioxidant activity, pressing yield, and average content of caffeic acid glucoside, quinic acid, kaempferol-3,7-di-*O*-glucoside, and chrysoeriol-7-*O*-apiosylglucoside were obtained in juices from peeled celery. Maceration of the pulp with amylase resulted in a significant reduction in antioxidant activity compared to control samples. An is-total increase of 17–41% in total flavonoid content was observed in all juices tested after treatment with the enzyme for 30 and 60 min, and the phenolic acid content increased by 4–41% after treatment of the pulp with amylase for 60 min. The 60 min holding of the pulp at 25 °C, including with the enzyme, was shown to decrease the antioxidant activity and the content of quinic acid, ferulic acid, and chrysoriol-7-*O*-apiose-glucoside in the juices tested compared to the samples held for 30 min, while the content of other phenolic acids and flavonoids increased. In addition, after 60 min of enzymatic maceration, the pressing yield of the juices increased.

## 1. Introduction

Celery root (*Apium graveolens*) is a vegetable in the family *Apiaceae*, rich in biologically active components [1,2]. Thanks to them, it exhibits anti-inflammatory, antiallergic, antioxidant, antimicrobial, and antiviral properties [3]. However, due to its specific organoleptic characteristics, it has not been found to be applicable in the fruit and vegetable industry, and its morphological structure significantly hinders the extraction of juice, affecting the profitability of its production. In order to increase juice yield and improve the sensory qualities or stability of the finished product, raw materials are subjected to enzymatic processing [3]. Enzymatic maceration of raw material pulp destroys cellular structures, reduces resistance to liquid flow, and extracts valuable biologically active components. This results in an increase in the nutrient density of the obtained products as well as an improvement in their organoleptic characteristics. According to Nowak and Tempczyk [4], enzymatic liquefaction of plant tissue, in addition to increasing the amount of pressed juice, also contributes to its enrichment in dyes, aroma compounds, pectin, and cellulose hydrolysis products, which are transferred to the liquid phase to a limited extent during traditional pressing.

Various types of enzymes are used to modify and improve the nutritional, functional, and sensory properties of finished products. They increase the rate of reactions and substrate transformations in biological material by up to 100–1000 times, directing their course [5]. Enzymes are obtained from natural sources, mainly 90% from microorganisms and plant and animal tissues. Microorganisms dominate amylase production due to their optimal growth requirements, high availability, efficiency, environmental friendliness, neutrality, and cost-effectiveness compared to amylases produced from plants or animals [6].

In the current food industry, amylases belonging to the glycohydrolase family are widely used [7]. Among them, α-, β- and γ-amylases can be distinguished, differing in the way in which they attack polysaccharide chains and in their sensitivity to pH and temperature. α-amylases catalyse the hydrolytic breakdown of α-1,4-glycosidic bonds, attacking bonds within the chain and forming low molecular weight dextrins [5]. Amylases are used to liquefy and saccharify starch, produce corn syrups, glucose–fructose syrups, and beer, soften dough or increase bread volume [8,9]. In the fruit and vegetable industry, they have been used to prepare jams and marmalades [10]. The use of these enzymes in the production of fruit and vegetable juices leads to the breakdown of pectin and starch [11], influencing the increase in pressing efficiency, improvement of the clarification process, and reduction of viscosity and turbidity in the beverages obtained [11,12]. Fungal and bacterial amylases can be distinguished among amylases. Fungal α-amylases, in contrast to bacterial α-amylases, show higher activity in environments with lower temperatures and lower pH, which should allow the production of high-quality final products and possibly with low degradation of bioactive components [5].

The purpose of this study was to determine the profile and content of phenolic compounds, antioxidant activity, as well as the pressing yield, extract content, and certain sugars in juices from the peeled and unpeeled pulp of root celery macerated with α-amylase from *Aspergillus oryzae* at the optimal of its action (25 °C, pH~6.0).

## 2. Results and Discussion

### 2.1. Contents of Certain Phenolic Acids in Root Celery Juices

Table 1 shows the content of phenolic acids identified in all celery juices. The juices showed the presence of caffeic acid glucoside, quinic acid, sinapoyl-caffeoylquinic acid, coumaroylquinic acid, and ferulic acid.

Caffeic acid glucoside in 100 mL of celery juice oscillated between 2.31 (AU1) and 9.46 mg (AU2). Under the influence of a longer duration of enzyme action in unpeeled celery root, a more complete maceration of the raw tissue may have occurred, and therefore, the bioavailability of the compound analysed increased; therefore, highly significant differences were shown between the results of AU1 and AU2. A higher caffeic acid glucoside content than juices of unpeeled celery (U) characterised juices made from peeled raw material (P). The application of an enzyme to celery pulp had a significant effect on the content of this compound. Its highest amount was determined in juices obtained from the peeled and unpeeled root pulp incubated with the enzyme for 60 min (AP2, AU2), and these values in 100 mL of juices were 7.06 mg and 9.46 mg, respectively. On the other hand, a shorter 30 min enzyme treatment in celery pulp (AP1, AU1), compared to control samples held at 25 °C for 0 and 30 min, resulted in significant reductions in caffeic acid glucoside content by an average of 53% and 31% in peeled celery juices, and by 62% and 48% in unpeeled celery juices. The duration of holding the celery pulp at 25 °C also proved to be an important factor. Its prolongation caused a decrease in the content of caffeic acid glucoside in juices pressed from peeled (P compared to P1 and P2) and unpeeled root pulp (U compared to U1 and U2) without the use of an enzyme Jaworska et al. [13] analyzed the effect of the addition of pectinase from *Rhizopus* sp. to celery pulp on the dioic acid content of the juice. The values ranged from 0.0 to 6.0 mg/L of juice, with the highest value for juice obtained from the peeled, non-enzymatically treated root. When the enzyme was added, the values were much lower. In a study by Nićetin et al. [14], of all the phenolic acids detected, caffeic acid was present in the celery root extract an amount of 55.46 mg/kg.

On the contrary, in the case of enzymatic maceration of peeled and unpeeled celery pulp, the relationship was the opposite. Pajević et al. [15] determined the caffeic acid content in celery roots at 2.3–3.9 μg/g of extract. Yao et al. [16] showed the presence of caffeic acid in the celery extracts tested at the level of 7.0–30.0 g/100 g d.m., and the lower of these values corresponded to the results obtained in our study. A study by Kaiser et al. [17] examined about 59.4 ± 1.2 mg/100 g d.m. of caffeic acid derivatives in root celery. The differences obtained between our own research and that of the authors (Kaiser et al. [17] and Pajević et al. [15]) are due to the different sample preparation for analysis and different test methods. For example, Kaiser [17] analysed celery pulps, which were made from blanched, ground, and reheated celery slices at 90 and 100 °C. In addition, these pulps were stored frozen until the analyses. In turn, HPLC performed analyses. Pajević et al. [15], on the other hand, used the LC-MS/MS method, and the samples were prepared as follows: 3× roots were macerated with methanol (6 h), the resulting filtrates were then evaporated on vacuum evaporators and then dissolved in DMSO.

The quinic acid content of the juices oscillated between 3.86 mg/100 mL (U2) and 9.59 mg/100 mL (AU1). Taking into account the method of root pretreatment, a significantly higher average quinic acid content was observed in juices of peeled celery than those obtained from unpeeled raw material. The highest amount of acid analysed was tested in juices pressed from unpeeled celery pulp macerated with α-amylase for 30 (AU1)—9.59 mg in 100 mL, and in the case of juice from peeled root pulp also incubated with the enzyme for 30 min (AP1) (7.26 mg/100 mL). Increasing the maceration time from 30 to 60 min resulted in a significant reduction in the content of quinic acid by 9.6–13% in all samples analysed, P2 to P1 by 10.6%, AP2 to AP1 by 13%, U2 to U1 by 9.6% and AU2 to AU1 by 10.4%. These values were higher compared to control samples held at 25 °C for the same time, without enzyme, by 53 and 16%, respectively. The longer duration of holding the peeled and unpeeled pulp at 25 °C without enzyme adversely affected the content of the analysed acid. According to Kaiser et al. [17], quinic acid derivatives are about 10 mg/100 g d.m. in celery root. Similar results of quinic acid content were obtained by Jaworska et al. [13], in celery juice. The values ranged from 43.7 to 139.5 mg/l of juice, with the highest levels obtained in juices pressed from celery pulp treated with the enzyme pectinase.

Sinapoyl-caffeoylquinic acid was also identified in celery juices, whose content ranged from 1.58 (P2) to 6.25 mg/100 mL (U1). Its highest amount was found in juices pressed from the unpeeled root. The application of the enzyme to the pulp of the peeled and unpeeled root had no significant effect on the level of the acid analysed, except for the juice of unpeeled celery macerated with α-amylase for 30 min (AU1). The sinapoyl-caffeoylquinic acid in this juice decreased by 35% compared to the control sample (U).

Coumaroylquinic acid was also identified in celery juices, whose content oscillated between 2.30 (AP1) and 7.84 mg (AP2) per 100 mL of product. A significantly higher average content was examined in juices from the unpeeled root. The maceration of the enzyme of the celery pulp significantly affected the content of coumaric acid in the juices tested. A 30 min incubation of the pulp obtained from the peeled root (AP1) resulted in a significant reduction in the content of coumaroylquinic acid of 46% compared to the control sample (P) and 66% compared to the control sample held at the same temperature for 30 min (P1). However, extending the maceration time from 30 to 60 min (AP2) resulted in a nearly 2-fold increase in the level of coumaric acid in the tested juice compared to the control sample (P). Particularly noteworthy is the increase in Coumaroylquinic acid content when maceration time was extended in AP1 and AP2 samples. Analysing the effect of holding time of celery pulp at 25 °C, it was noted that its prolongation contributed to an increase in the content of coumaroylquinic acid in the juices, except for the control samples from the unpeeled root, where the relationship was the opposite (U, U1, and U2).

The ferulic acid content of the celery juices tested was 1.31–8.09 mg/100 mL. Significantly higher average results were obtained in juices formed from the unpeeled root. In the macerated juice of the peeled root for 30 min with α-amylase (AP1), the content of ferulic acid was three times higher than in the control juice (P1). It is noteworthy that holding the P2 and U2 samples for 60 min at 25 °C resulted in a highly significant reduction in ferulic acid content compared to the control sample. This ferulic acid status is probably due to the fact that ferulic acid (FA) affects the formation of certain Maillard reaction products, i.e., early MRPs, fluorescent and nonfluorescent advanced glycation end products (AGEs), and melanoidins in model systems [18]. Analysing the results obtained after enzymatic maceration of the pulp of the unpeeled root, it was observed that compared to the control sample (U), the ferulic acid content decreased by 32% (AU1) and by 36% (AU2). However, comparing the ferulic acid content with the control samples, held at 25˚C for the same time (U1 and U2), the application of the enzyme to the pulp from unpeeled raw material resulted in a higher content level of the analysed compound in the tested so-kids. The duration of holding the celery pulp at 25 °C also proved to be an important factor. It was noted that its prolongation had an unfavourable effect on the content of the analysed compound in all juices studied. In a study by Pajević et al. [15], the content of ferulic acid in celery roots oscillated between 9.5 and 29.0 μg/g of dry extract. Yao et al. [16] determined the content of this acid in 11 celery extracts at the level of 11–93 mg/100 g d.m., depending on the variety of raw materials. A study by Kaiser et al. [17] indicated that the content of ferulic acid derivatives in root celery was about 2.7 mg/100 g d.m. Much lower results than those obtained in our own study were obtained by Jaworska et al. [13], who determined the ferulic acid content of unpeeled root juice after the addition of pectinase from 2.2 to 2.5 mg/L for peeled root juice from 9.4 to 9.6 mg/L, and these values were lower compared to control samples. The differences between our own studies and those obtained by the aforementioned authors may have been due to the fact that different enzymes were acting on the celery pulp, at a different dose, and may also have been affected by the timing of obtaining the raw material for the study. In a study by Nićetin et al. [14], of all the phenolic acids detected, ferulic acid was present in the celery root extract at an in the amount of 11.09 mg/kg. The total amount of phenolic acids in the celery juices studied ranged from 21.1 (U2) to 35.5 mg/100 mL (AU2) and was expressed as the sum of caffeic acid glucoside, quinic acid, sinapoyl-caffeoylquinic acid, coumaroylquinic acid, and ferulic acid. The significantly higher average total content of the compounds analysed was found in the juices of the unpeeled root, except for the samples marked P2 and U2, for which no significant differences were created. Maceration of α-amylase peeled pulp for 30 min (AP1) had no significant effect on the sum of phenolic acids in the juices tested, compared to the control sample held at 25 °C for the same time (P1). The incubation of peeled and unpeeled pulp with the enzyme for 60 min (AP2 and AU2) significantly increased the total sum of phenolic acids in the juices by 4% and 7%, respectively, compared to control samples (P and U). These samples also had 37% and 68% more total polyphenols, respectively, compared to control samples held for 60 min at 25 °C (P2 and U2). The time of holding the pulp at 25 °C was also an important factor in determining the content of total phenolic acids in the juices. With its elongation, the total sum of phenolic acids in juices squeezed from peeled and unpeeled pulp decreased (samples P1 I P2 compared to P, and samples U1 I U2 compared to U), while it increased in juices from enzyme-treated peeled and unpeeled pulp (comparing samples AU1 to AU2 and AP1 to AP2). The total phenolic acid content expressed as the sum of caffeic, ferulic, and p-coumaric acids in celery extracts studied by Yao et al. [16] was 113–223 mg/100 g d.m. According to Arsenov et al. [19], among phenolic acids in celery roots, ferulic acid was dominant, followed by chlorogenic acid and caffeic acid, and among flavonoids in celery root, apigenin was dominant.

Phenolic compounds have different amounts of hydroxyl groups and different polymeric forms, making them easily degraded or oxidised [20]. It has also been found that various thermal treatments, i.e., boiling, blanching, or drying, can improve the antioxidant properties of many compounds. As mentioned above, these treatments can break down the glycosidic forms of flavonoids or reduce the phenolic content during changes in their matrix constituents [1]. Vagiri and Jensen [21] further demonstrated that mild heating at 40–50 °C increases the extractability of bioactive components in products. This is due to the release of compounds from the plant chromoplasts, which results in an increase in their total concentration in the finished product. Taking into account the above reports, it can be concluded that the low holding temperature of celery pulp, as well as the relatively short maceration time with the enzyme (30–60 min), may have influenced the release of phenolic compounds in the pulp and allowed their passage into solution (juice). Yao et al. [16] and Wojdylo et al. [22] showed that caffeic acid exhibits high antioxidant activity because it has a 3,4-dihydroxylation position on the fenol ring. Moreover, it has an additional coupling in the propene side chain, which facilitates electron delocalisation through resonance between the propene group and the aromatic ring. Part of the celery root pulp was subjected to enzymatic hydrolysis, which results in specific bond breaking and deglycosylation of phenolic compounds. On the other hand, the degradation of phenolic acids can be explained by the action of oxygen and light, which can lead to the formation of simple or complex forms of phenols and other metabolites, with a consequent reduction in the content of individual compounds [1].

### 2.2. Contents of Certain Flavonoids in Celery Root Juices

In the study, the following flavonoids were identified in celery juices: Apigenin, apigenin-6-*C*-glucoside, acetylated apigenin-*C*-hexoside-*O*-pentoside, kaempferol- 3,7-di-*O*-glucoside, chrysoeriol, chrysoeriol-7-*O*-apiosylglucoside, chrysoeriol-7-*O*-6″-malonyl glucoside, chrysoeriol-7-*O*-glucoside, luteolin-7-*O*-glucoside, luteolin-7-*O*-malonyl-apiosylglucoside B, and acetylated luteolin hexoxyl-rhamnoside. Among the identified flavonoids, five compounds were quantified, the content of which is shown in Table 2. The content of apigenin 6-*C*-glucoside in the tested juices ranged from 0.85 (U2) to 2.68 mg/100 mL (AU2). In the case of samples not treated with an enzyme, slightly higher values were obtained in juices obtained from peeled celery pulp (P1, P2) than unpeeled (U1, U2). Adding enzyme to the pulp of the peeled root resulted in a significant decrease in the content of the compound analysed (AP1, AP2), while enzymatic maceration of the pulp of the unpeeled root increased it (AU1, AU2). The highest levels of apigenin 6-C-glucoside were examined in juices pressed from unpeeled root pulp macerated with α-amylase for 30 min (2.57 mg/100 mL, AU1) and 60 min (2.68 mg/100 mL (AU2). It was also observed that prolonging the time the pulp was kept at 25 °C contributed to a significant reduction in apigenin 6-C-glucoside content in control juice samples (P and U) from peeled and unpeeled roots. The content of apigenin-6-C-glucoside in celery juice obtained from the pulp of the unpeeled and peeled root after pectinase treatment was 0.2 to 1.2 mg/L and was highest in juices from the unpeeled root [13]. Different results may indicate the use of different enzymes, but also, for example, different agrotechnical conditions of cultivation of these plants, and the manner and timing of harvesting, which may translate into the level of analyzable compounds.

Also identified in the juices was kaempferol 3,7-*O*-diglucoside, whose content in the juices studied ranged from 0.25 (U) to 0.88 mg/100 mL (AP2). Its content was comparable to juices pressed from the pulp of the peeled and unpeeled root. Enzymatic maceration of the pulp significantly affected the average content of the analysed compound in the juices studied. The highest values of kaempferol 3,7-*O*-diglucoside were found in juices pressed from the pulp of peeled and unpeeled celery inoculated with enzyme for 60 min, 0.88 mg (AP2), and 0.77 mg/100 mL (AU2), respectively. These values were 42% and 35% higher, respectively than the same samples held at 25 °C for 60 min (although no statistical significance was found in the second case). However, it was also noted that as the time of holding celery pulp at 25 °C increased, the content of the analysed compound also increased in the control samples. According to Yao et al. [16], the kaempferol content of celery ranged from 0.6 to 3.3 mg/100 g d.m., and significant differences between different samples were likely due to genotypic and environmental differences, including location, UV-B radiation, disease and pest exposure, choice of parts tested and sampling time.

Among the flavonoids found in celery juices, apigenin was also found. Its content oscillated between 0.98 (U) and 2.84 mg/100 mL (AU2). The peeling of the celery root significantly affected the content of this compound in the juices, and higher average values were observed in the juices pressed from the pulp of the unpeeled root (U1, U2). Applying the enzyme to the unpeeled root pulp (AU1, AU2) did not have a significant effect on the apigenin content of the juices tested compared to control samples held for the same period (U1 and U2). However, when comparing the juice of AU1 and AU2 with the control sample (U), a significant, more than twofold increase in the content of the compound analysed compound in the juices studied. The addition of enzyme to the peeled pulp (AP1 and AP2) had an unfavourable effect on the value of apigenin in celery juices compared to control samples kept for the same time at 25 °C (P1 and P2), while in comparison to the control sample (P), the opposite relationship was found. The processing time of celery pulp at 25 °C was a significant factor affecting the apigenin content of the juices. In all the juices analysed obtained from the enzyme macerated pulp, a beneficial effect of a longer time to keep the celery pulp at 25 °C was observed (AP2, AU2), as, after 60 min of maceration, there was 25–35% more apigenin than after 30 min of enzymatic treatment. Pajević et al. [15] showed apigenin content ranging from 0.25 to 4.01 μg of quercetin equivalent/g of dry celery root extract, and the discrepancy in results was due to the derivation of raw materials from different locations.

On the other hand, according to Lim [23], the apigenin content of the root celery is approximately 2.4 mg/100 g, which coincides with the results obtained in our study. These results are also confirmed by Lugast and Hóvári [24], who put the apigenin content of celery root juices at 24 mg/kg bw. In the d.m. of celery, the content of apigenin was determined to range from 55 to 143 mg/100 g, and these values varied depending on the variety of raw materials used [16].

Chrysoeriol was also quantified in celery juices. Its average value in the samples ranged from 0.93 (AP1) to 2.77 mg/100 mL (U2). Significantly higher average values were observed in juices from the unpeeled root, and the highest chrysoeriol content was recorded in the juices, which were pressed from the pulp of the unpeeled root and incubated at 25 °C for 60 min (U2). On the contrary, a 30 min α-amylase treatment of unpeeled celery pulp (AU1) increased the chrysoeriol content of the juice by approximately 20% compared to a control sample held at the same temperature and time (U1). It was also found that the longer the time the pulp was kept at 25 °C, the higher the chrysoeriol content in all the celery juices tested. The chrysoeriol content of celery root, according to Pajević et al. [15], was 0.19 to 2.01 μg/g of dry extract.

The last flavonoid in celery juices, tabulated in the table, was chrysoriol-7-*O*-apiose-glucoside. Its content in the juices ranged between 0.12 (P1) and 2.07 mg/100 mL (AP1), with higher average values recorded in the juices made from the peeled root. At the same time, it is worth noting that juices from unpeeled and nonenzymatically treated pulp had the compound in question more. Enzymatic maceration of peeled celery pulp contributed to a significant increase in the content of chryzeoriol-7-*O*-apiose-glucoside in the juices, and the values were 2-fold to as much as 17-fold higher compared to control samples kept at 25 °C for 0, 30, and 60 min. In the case of unpeeled celery, a reduction in the amount of the compound in question was observed in juices from enzyme-treated pulp compared to the control sample (U). The duration of holding the celery pulp at 25 °C was also an important factor in determining the za-value of this compound in the juices. With its increase, the content of chryzeoriol-7-*O*-apyosylglucoside in the tested samples significantly decreased.

The flavonoid content in the studied juices oscillated between 5.50 (P)-9.05 mg/100 mL (AU2). Peeling the celery root significantly affected the average content of the compounds analysed in the juices. On average, juices obtained from unpeeled roots were characterised by a higher total flavonoid content compared to juices pressed from peeled celery pulp. The application of enzyme to the pulp of peeled and unpeeled roots significantly increased the flavonoid content of the juices analysed. Incubation time with the enzyme did not significantly affect the average total flavonoid content.

Nevertheless, the 30 min maceration of the peeled root pulp (AP1) increased the total of these compounds in the juice by nearly 25% compared to the control sample (P) and by 21% compared to the control sample held at 25 °C for 30 min. The longer incubation time of the peeled pulp with α-amylase (AP2) also increased the flavonoid content of the juices by 31% and 28% compared to the control samples (P) and (P2) samples. In juices pressed from unpeeled root pulp macerated with the enzyme for 30 min (AU1), an increase in the amount of total flavonoids was observed by 59% and by 24% compared to control samples (U) and (U1). On the other hand, the enzyme treatment of unpeeled pulp for 60 min (AU2) contributed to an increase in the level of flavonoids in the juices by 71% compared to the control sample (U) and by 28% compared to the control sample (U2). According to Yao et al. [16], in celery, the total content of flavonols, which included apigenin, luteolin, and kaempferol, oscillated between 85 and 175 mg/100 g d.m., and the significant differences between the samples studied were probably due to genotypic and environmental differences. According to Popova et al. [25], the total flavonoid content of celery roots is about 1.0–4.8 mg quercetin equivalent/g d.m., and according to Lugast and Hóvári [24], there is about 26 mg/kg of them in celery root. Liao et al. [26] point to the desirability of using enzymes to increase the levels of health-promoting compounds. They report that enzymatic treatment of carrots increased the carotenoid content of carrot juice by nearly 1.5 times compared to a control, untreated sample.

The basic structure of flavonoids is a flavin nucleus consisting of 15 carbon atoms arranged in 3 rings, designated A, B, and C. Differences in structure and ring substitution affect the antioxidant potential of these compounds [22]. The antioxidant activity of flavonoids is dependent on the structure and sub-substitution pattern of the hydroxyl groups, and the prerequisite for effective free radical scavenging is the 3,4-ortho-dihydroxy configuration in the B ring and the C ring the 4-carbonyl group. The presence of the double bond C2–C3, which is configured in the 4-keto arrangement, is responsible for the delocalisation of electrons from the B ring, which, in consequence, increases free radical scavenging activity [16,22]. Apigenin and kaempferol have the same number of hydroxyl groups, with 4- and 5,7-dihydroxy groups in rings B and A, respectively. Given that apigenin is the predominant flavonoid in celery, it may be mainly responsible for the antioxidant activity of flavonoids [16]. In our study, we found that significantly higher antioxidant activity as measured by ABTS˙^+^ and DPPH˙ assay was observed in juices from peeled pulp macerated with enzyme for 30 min, and this juice also had higher levels of total flavonoid content compared to control samples (P, P1 and P2).

### 2.3. Antioxidant Properties and Total Polyphenol Content of Root Celery Juices

Table 3 shows the results of antioxidant potential and total polyphenol content in the celery juices obtained. The antioxidant activity of the juices measured by the ABTS*^+^ method oscillated between 353 (AU2) and 510 µmol Trolox/100 mL (AP1). Analysing the results, a significant effect of pretreatment of celery root and the time of holding the pulp at 25 °C on the antioxidant characteristics of the tested juices was observed. Significantly higher values, on average by 4% for samples P and U up to 10% for samples P1 and U1, were obtained in juices pressed from peeled celery roots compared to juices from unpeeled roots. The application of enzyme to celery pulp did not significantly affect the average value of the parameter studied.

Nevertheless, considering the interaction of the experimental factors, in the case of juice obtained from peeled celery pulp macerated for 30 min with α-amylase (AP1), the antioxidant activity was significantly higher compared to the control samples (P and P1) and amounted to 510 µmol TE/100 mL. On the other hand, after 30 min of maceration with the enzyme, the unpeeled celery showed a significant reduction in the activity in question compared to the control sample (U) and no significant differences with respect to the control sample (U1). It was also noted that with increasing the time the pulp was kept both peeled and unpeeled at 25 °C, the antioxidant activity measured by the ABTS*^+^ method decreased by 14–20%. According to Yao et al. [16], antioxidant activity measured by the ABTS*^+^ method in 11 celery varieties ranged from 81.9 to 114.4 µmol TE/100 g d.m., indicating that root celery was more active than celery with respect to the ABTS*^+^ cation radical. Similar results to the values obtained in our study were obtained by Yao and Ren [27], who analysed the antioxidant activity using the same test. They indicated that the antioxidant potential of celery was in the range of 585–835 µmol TE/100 g of fresh weight. Priecina et al. [1] suggested that the antioxidant activity of celeriac after peeling, in the ABTS*^+^ test, was about 136 µmol TE/100 g d.m., so these values were about 3.5 times lower than those obtained in the work in question for peeled celery juice. Similar results were obtained by Jaworska et al. [13] investigating antioxidant activity with the ABTS*^+^ assay in celery juices, and the values ranged from 3666–4759 μmol Trolox/L. The highest values were recorded in juices squeezed from peeled and unpeeled celery, without enzyme, and in juice from the peeled root macerated for 1 h with pectinase.

The results of DPPH* radical activity in celery juices ranged from 37 (AU2) to 74 µmol TE/100 mL (AP1). Similarly, as for the analysis of antioxidant potential measured by the ABTS*^+^ method, it was noted that significantly higher DPPH* radical activity values were obtained in juices made from the peeled root. Maceration of the peeled celery pulp with the enzyme for 30 min (AP1) contributed to an average increase of 8% and 35% in the antioxidant activity of the juice compared to the control sample held for 0 (P) and 30 min (P1), respectively, at 25 C. In contrast, a 60 min α-amylase treatment of unpeeled celery pulp (AU2) reduced the antioxidant potential of the juice by 29% compared to the control sample held for 0 min at 25 °C (U). Furthermore, it was observed that the antioxidant activity of juices pressed from celery pulp decreased when held at 25 °C for a longer period of time (60 min) compared to those held for 0 and 30 min, except for samples P2 to P1. Yao et al. [16] also tested the antioxidant activity of different varieties of celery. The DPPH* method obtained values oscillating between 79.5–105.8 μmol TE/100 g d.m. Yao and Ren [27] determined significantly higher results of DPPH* free radical scavenging capacity in celery at the level of 612–734 μmol TE/100 g d.m. In peeled celery, Priecina et al. [1] reported an antioxidant potential measured by the DPPH* assay of 126 μmol TE/100 g d.m., which was about two times higher than in the present study. According to Jaworska et al. [13] the DPPH* radical scavenging activity in celery juices ranged from 449 to 1108 µmol Trolox/L and was highest in juices from celery pulp macerated with pectinase. Enzymatic maceration of celery pulp peeled for 30 and 60 min contributed to an increase in the antioxidant capacity of the juices by 57% and 31%, respectively, compared to control samples stored at 25 °C for the same time.

The total polyphenol content in the juices tested oscillated between 12 (P2) and 22 mg GAE/100 mL (U). The juice from the unpeeled root held at 25 °C for 0 min (U) and after its 30 min enzymatic maceration (AU1) had the highest polyphenol content. Juices pressed from the unpeeled root were characterised by a higher content of total polyphenols than juices made from the pulp of the peeled root. The enzyme maceration of the peeled celery pulp had no significant effect on the level of the parameter analysed, while a significant difference was observed in the juice of the unpeeled raw material after 30 min of incubation with the enzyme (AU1). Popova et al. [25] determined the content of polyphenolic compounds in celery root extract at about 8.2–13.6 mg GAE/g d.m. In celery root (Egor and Dobrynya) according to Golubkina et al. [28] the content of polyphenols is about 10.8 mg GAE/g d.m. The differences in the study may be due to different sample preparations (celery corns were homogenized, dried at 70 °C, and then methanol extracts were prepared from them), as well as different test methodology. In contrast, Pajević et al. [15] indicated similar values oscillating between 5.0–8.5 mg GAE/g dry extract. In a study by Yao et al. [16] in different varieties of celery, the content of total polyphenols ranged from 3.5 to 5.0 mg GAE/100 g d.m. In addition, Kaiser et al. [17] studied the polyphenol content of the celery root paste, indicating values in the range of 1.2 g GAE/kg d.m. A study by Jaworska et al. [13] showed that the total polyphenol content of celery juices ranged from 93 mg to 225 mg GAE/L. The addition of pectinase and incubation time in celery pulp had a statistically significant negative effect on the total polyphenol content of the juices studied. Salamatullah et al. [29] revealed that after heat treatment, the polyphenol content in 100 g celeriac decreased from 22.2 mg GAE in control to 3.0 mg GAE. The accumulation of polyphenols in a plant varies depending on the genotype, the part of the plant, agroclimatic conditions, as well as the timing of harvesting and post-harvest processing [30].

### 2.4. The Yield of Pressing, Total Extract, and Total Sugars in Root Celery Juices

Table 4 shows the results of pressing yield, total extract content, and total carbohydrate content (fructose, glucose, and sucrose) in the obtained celery juices. Juice pressing yields ranged from 47.3% (P) to 72.3% (AP2) and were lowest for control samples, without enzyme, with zero incubation time at 25 °C (P and U). Analysing celery root treatment, it was observed that significantly more juice was extruded from the peeled pulp (P1, P2) than from the unpeeled pulp (U1, U2). A 30 min maceration of the peeled pulp with α-amylase (AP1) compared to the control sample held at 25 °C for the same period of time (P1) resulted in a 17% reduction in yield. In contrast, an hour-long enzyme treatment in peeled (AP2) and unpeeled (AU2) pulp effectively increased juice yields by nearly 53–55% compared to untreated control samples (P) and (U) and by 8% and 18% compared to control samples held for the same time at 25 °C (P2) and (U2), respectively. Considering the storage time of the celery pulp at 25 °C, it was noted that with increasing storage time, the pressing efficiency increased in all samples analysed, including after enzymatic maceration of the pulp, with this increase not statistically proven for samples P1 and P2. In Nadulski’s study [3], the yield of pressing the juice of the celery root pulp ranged from 28 to 54%, while after freezing the celery pulp, thawing it, and pressing the juices, its yield increased to as much as 75%. Similar high results were obtained in our research during, i.e., 60 min enzymatic maceration, and therefore, in a much shorter time than the time required to freeze the pulp, thaw it, and squeeze the juice. Furthermore, it should also be mentioned that the efficiency of pressing root vegetables, including celery, is influenced by the degree of crushing of the raw material, its temperature, and the selection of the appropriate enzyme treatment [31,32]. Liao et al. [26] analysed the effect of macerating carrot pulp with the Pectinex Smash XXL enzyme product, showing that it has a beneficial impact on juice pressing yield, causing a 20% increase in juice yield compared to the control sample. In our study, an increase in yield was observed after enzymatic ob-treatment with amylase, which could be due to polysaccharide degradation, which, as suggested by Abdullah et al. [33], has the effect of reducing viscosity, and this facilitates the process of centrifugation, filtration or just pressing.

Analysis of the extracted content of the juices showed that the method of pretreatment of the root, as well as the interaction between the method of treatment and the enzyme, had a significant effect on the parameter analysed. It was shown that significantly higher extract content was obtained in juices from unpeeled pulp, where the average extract content was 8.03 (U1)–8.27°Bx (U), than in juices pressed from the peeled pulp (6.90 (P)–6.87°Bx (P2)). Adding α-amylase to the peeled pulp increased the extract in the juices by 9–16% and decreased the extract in the juices from the unpeeled pulp by 9–13% compared to the control samples, but these changes were not statistically significant. The time the pulp was kept at 25 °C did not significantly affect the extracted content of all pressed juices. In the Nadulski study [3], the extract content of the juices extruded from celery pulp oscillated between 8.3 and 9.1°Bx, and these values were similar to Nadulski’s own results obtained for juices from the unpeeled root. Nadulski [3] also checked the extracted content of the juices made of the unfrozen celery pulp of the chips, where the values were lower, around 6.3°Bx. In addition, in another publication, Nadulski et al. [34] found that the juice obtained from fresh carrot roots had several % lower extract content than the juice obtained from frozen and thawed pulp of chips.

The total sugar content in 100 mL of the juices tested, which was the sum of glucose, fructose, and sucrose, ranged from 3.20 (AP2) to 5.16 g (U1). Significantly higher values, on an average of 6–22%, were obtained in juices made from unpeeled pulp than from peeled pulp. The addition of enzyme contributed to a slight reduction in sugars in juices of unpeeled pulp (AU1, AU2) by 1–17% and in celery juice peeled after 60 min of maceration (AP2) by 19–25% compared to control samples. The 30 min enzyme action in the peeled pulp (AP1) resulted in a slight increase in carbohydrate levels by 8–10% compared to the control samples (P and P1). Analysing the effect of the holding time of peeled and unpeeled celery pulp at 25 °C on the sugar content of the juices, it was observed that compared to the control samples (P and U), both 30 and 60 min resulted in a slight increase, except for the juice of the unpeeled pulp held for 60 min at the identical temperature (AP2). According to Lim [23], there are about 9 g of carbohydrates and about 1.6 g of total sugars in 100 g of an edible serving of root celery. However, the sugar content in different vegetable raw materials depends on the variety and changes during ripening and storage. Hence, there may be differences in their content. Jaworska et al. [13] showed that juices from unpeeled celery contained significantly more fructose and glucose, while sucrose dominated in juices pressed from peeled celery. In terms of sugar content, 1 L of celery juice contained: 2.8–6.3 g of fructose, 14.9–25.0 g of glucose and 15.7–22.1 g of sucrose. The highest sucrose content in the juice was found after 60 min incubation of peeled celery pulp at 25 °C and after maceration of the pulp with pectinase.

## 3. Materials and Methods

### 3.1. Plant Material

The material for the study was celery (*Apium graveolens* L.) roots of the *Zagloba* variety purchased from a local wholesaler 50°0′12.5892″ N and 22°1′44.8392″ E (Rzeszow, Poland). Raw materials with a diameter of not less than 6 cm were used for the study. Celery was characterised by its spherical shape, cream-grey colour, intense aroma and taste, and absence of mechanical and microbiological damage. The roots were processed into juice immediately after purchase.

### 3.2. Methods

#### 3.2.1. Celery Juice Production Technology

The peeled and unpeeled celery roots were sorted, washed, and ground into a pulp using a Vorwerk-Thermomix TM31 multifunctional device (Vorwerk, Wuppertal, Germany). The pulp obtained from peeled and unpeeled celery was divided into five parts of 300 g each. Due to the pH of the celery pulp oscillating around a value of 6.0, it was not adjusted before the enzyme was administered. To the four pulps, from both peeled and unpeeled celery, 45 mg of α-amylase from *A. oryzae* was added and incubated at 25 °C for 30 and 60 min. Control samples were prepared from peeled and unpeeled celery, without the addition of the enzyme, and held at 25 °C for 0 min, 30 min, and 60 min. After the set time, the juice was extruded from the pulp using a Norwalk Juicer type 275 hydraulic press (Seattle, WA, USA).

An α-amylase isolated from *A. oryzae* was used to macerate celery pulp. The enzyme has an activity of ~30 U/mg and is in a dry, powdered form. Due to the removal of a significant amount of water, it has a higher shelf life and increased unit activity compared to liquid enzymes.

#### 3.2.2. Determination of the Content of Certain Phenolic Compounds in Juices

Before analysis, the juices were centrifuged in a laboratory centrifuge MPW-260R (MPW Med. Instruments, Warsaw, Poland) at 7300 RPM for 5 min and cleaned using PTFE syringe filters with a pore size of 0.45 µm. Determination of compounds was performed using an ACQUITY UltraPerformance LC^®^ Liquid Chromatograph (UPLC-PDA-MS/MS) (Waters Corporation, Milford, MA, USA) coupled to a photodiode array (PDA) detector and a tandem, dual quadrupole mass detector (TQD) (Waters ACQUITY^®^ TQD (Tandem Quadrupole Detector), (Micromass, Wilmslow, UK) with an electron sputter ionisation (ESI) source. The following TQD parameters were used: Cone voltage 30 V, capillary voltage 3500 V, source temperature 120 °C and desolvation temperature 350 °C, and nitrate (desolvation gas) flow rate 800 L/h. Separation of phenolic compounds was carried out using a BEH RP C18 UPLC column (1.7 µm, 2.1 mm × 100 mm, Waters Corporation) at 50 °C ± 5 °C. The phase consisted of solvent A (0.1% aqueous solution of formic acid, *v*/*v*) and solvent B (40% aqueous solution of acetonitrile, *v*/*v*). The flow rate was kept constant at 0.3 mL/min, and the per-injection volume was 5 µL. All determinations were performed in triplicate.

Calculation of the compactness of phenolic acids was made on the basis of calibration curves (for ferulic acid, caffeic acid, and coumaric acid), the dependence of the peak area on the concentration of the substance injected into the column. The standard substance for the determination of flavonoid content was vitexin. Results are given as average values from three replicates performed in the unit mg per 100 mL of juice.

#### 3.2.3. Determination of Antioxidant Potential of Juices

The juices were centrifuged in an MPW-260R laboratory centrifuge (MPW Med. Instruments, Warsaw, Poland) at 7300 rpm./min for 10 min before analysis of antioxidant potential.

The antioxidant potential measured by the ABTS˙^+^ method was performed according to Re et al. [35]. The decanted juices were appropriately diluted with methanol and destined for analysis. The cation radical ABTS˙^+^ was generated by chemical oxidation with K_2_S_2_O_8_. 0.00195 g of ABTS˙^+^ and 0.0033 g of K_2_S_2_O_8_ were weighed in 7 cm^3^ of phosphate buffer (pH = 7.4). The resulting ABTS˙^+^ cation radical solution was incubated for 16 h in a dark place at room temperature. The prepared radical was diluted with deionised water so that at λ = 734 nm, the absorbance was 0.700 ± 0.020 nm. Into 3.5 mL quartz cuvettes were added 30 μL dilutions of the test samples and 3 mL of ABTS˙^+^ cation radical solution. The prepared samples were left in the dark for 6 min. The reference sample was made in the same way, replacing the Trolox solution with distilled water. After time had elapsed, the absorbance of the samples was measured at λ = 734 nm against distilled water using a UV-1900 UV-Vis spectrophotometer (Shimadzu, Kyoto, Japan). The results of the determinations are given in terms of μmol Trolox equivalent (TE) in 100 mL of juice.

Free radical scavenging capacity was performed using the method operated by Yen and Chen [36] after the juices had been prepared according to the method described above. To 3.5 mL quartz cuvettes, 500 μL dilutions of the test samples and 2 mL of DPPH radical solution were added. The prepared samples were left in the dark for 10 min. The reference sample was made in the same way as the Trolox solution, replacing it with 96% methanol. After the time had elapsed, the absorbance of the samples was measured at λ = 517 nm against methanol. All determinations were performed in triplicate, and the final re-490 results were presented in μmol TE/100 mL of juice.

The total polyphenol content of the juices was determined using the method developed by Xianggun et al. [37]. Into 3.5 mL quartz cuvettes were added 0.1 mL of the prepared solutions of the test samples, 2 mL of distilled water, 0.2 mL of Folin–Ciocalteau reagent, and 1 mL of 20% aqueous sodium carbonate solution. The prepared samples were left in the dark for 1 h. After that time, the absorbance of the samples was measured at λ = 765 nm against distilled water. From the results obtained, the sum of polyphenols in the samples was calculated using a calibration curve. All determinations were performed in triplicate, and results were presented in mg of gallic acid equivalent (GAE) in 100 mL of juice.

#### 3.2.4. Determination of Pressing Yield, Extract and Total Sugars in Juices

The juice yield was calculated using the formula: Wj = M/Mp × 100, where: Wj—yield of the pressing process [%], M—the weight of juice obtained during pressing [g], Mp—the initial weight of pulp used for pressing [g]. The results are presented as average values from the three replicates performed in %.

Total extract content was determined using an Abbe Zuzi refractometer, type 325, according to PN-EN 12143:2000 [38]. The measurement was performed at a temperature of 20 ± 1 °C, and the result, presented as the average value of the three measurements obtained, was given in °Brix.

HPLC performed the carbohydrate content, and samples for determination were prepared according to the method described in Section 3.2.2. A high-performance liquid chromatograph HPLC from SYKAM (SYKAM, Fürstenfeldbruck, Germany) controlled by Clarity Software version 6.1 (UK) was used for the analysis. Separation was carried out on a Cosmosil SUGAR-D column (4.6 mm × 250 mm), and elution was carried out at a flow rate of 1 mL/min, pressure max. 100 Ba and a temperature of 35 ± 5 °C. The injection volume was 20 µL. The mobile phase consisted of acetonitrile (phase A: 75%) and water (phase B: 25%). Quantitative analysis was performed using a standard curve for fructose, glucose, and sucrose as standard substances. The analysis was performed in triplicate for each juice sample, from which the arithmetic mean was drawn. The results are presented as the total content of fructose, glucose, and sucrose in g per 100 mL of juice.

### 3.3. Statistical Analysis of Results

Statistical analysis of the results was done using the Statistica ver. 13.3. (TIBCO Software Inc., Palo Alto, CA, USA) [39]. All analyses were performed in triplicate, and results were expressed as the mean ± SD. The results were subjected to a three-factor analysis of variance (ANOVA), finding significance at *p* < 0.01 and a between-groups MANOVA. The significance of differences between averages were verified by the Duncan test at the significance level *p* < 0.01.

## 4. Conclusions

Both peeling of the raw material, maceration of the pulp with α-amylase, and the time of keeping the celery pulp at a temperature of 25 °C had a significant impact on the profile of phenolic and antioxidant compounds in the vast majority of the analysed cases. activity and other parameters analysed in celery juices. It was found that juices from unprocessed raw materials were characterised by a higher average content of phenolic acids and flavonoids, total polyphenols, and sugars. Maceration of celery pulp with α-amylase significantly increased the total content of flavonoids in the juices compared to control samples, while the total content of phenolic acid was higher only in juices from the pulp after 60 min of maceration with the enzyme. It was shown that a long time of keeping the pulp at a temperature of 25 °C, together with the enzyme, reduced the antioxidant activity and the content of apigenin 6-C-glucoside, ferulic acid, and quinic acid while increasing the content of other phenolic acids and flavonoids in the juices and increased the efficiency of juice pressing.

## Figures and Tables

**Table 1:** Content of certain phenolic acids in celery juices.

Sample Name	Caffeic Acid Glucoside [mg/100 mL]	Quinic Acid [mg/100 mL]	Sinapoyl-Caffeoylquinic Acid [mg/100 mL]	Coumaroylquinic Acid [mg/100 mL]	Ferulic Acid [mg/100 mL]	Total Phenolic Acids [mg/100 mL]
P	8.91 ^g^ ± 0.09	7.10 ^e^ ± 0.06	2.73 ^bc^ ± 0.02	4.25 ^b^ ± 0.03	5.93 ^g^ ± 0.09	28.90 ^de^ ± 0.20
P1	6.08 ^e^ ± 0.05	6.82 ^d^ ± 0.09	2.67 ^b^ ± 0.03	6.81 ^e^ ± 0.07	2.41 ^c^ ± 0.05	24.80 ^b^ ± 1.00
P2	5.89 ^e^ ± 0.03	6.10 ^c^ ± 0.02	1.58 ^a^ ± 0.08	7.03 ^e^ ± 0.01	1.31 ^a^ ± 0.01	21.90 ^a^ ± 0.20
AP1	4.17 ^c^ ± 0.10	7.26 ^e^ ± 0.02	2.65 ^b^ ± 0.02	2.30 ^a^ ± 0.04	8.09 ^h^ ± 0.08	24.50 ^b^ ± 1.00
AP2	7.06 ^f^ ± 0.04	6.30 ^c^ ± 0.07	2.92 ^c^ ± 0.06	7.84 ^g^ ± 0.11	5.85 ^g^ ± 0.02	30.00 ^e^ ± 0.10
U	6.15 ^e^ ± 0.03	6.56 ^cd^ ± 0.01	5.44 ^e^ ± 0.03	7.38 ^f^ ± 0.11	7.79 ^h^ ± 0.03	33.30 ^ef^ ± 0.00
U1	4.46 ^d^ ± 0.02	4.27 ^b^ ± 0.03	6.25 ^f^ ± 0.10	6.80 ^e^ ± 0.07	4.11 ^d^ ± 0.08	25.80 ^c^ ± 1.00
U2	3.59 ^b^ ± 0.01	3.86 ^a^ ± 0.01	6.03 ^f^ ± 0.02	5.76 ^c^ ± 0.09	1.82 ^b^ ± 0.02	21.10 ^a^ ± 0.10
AU1	2.31 ^a^ ± 0.05	9.59 ^g^ ± 0.12	3.55 ^d^ ± 0.05	6.19 ^d^ ± 0.19	5.32 ^f^ ± 0.03	27.00 ^d^ ± 0.20
AU2	9.46 ^h^ ± 0.14	8.59 ^f^ ± 0.04	5.59 ^e^ ± 0.07	6.85 ^e^ ± 0.05	4.98 ^e^ ± 0.04	35.50 ^f^ ± 0.30
Three-factors analysis of variance ANOVA *p* < 0.01						
Celeriac root	0.0000	0.0000	0.0000	0.0000	0.0000	0.0000
Enzyme	0.0000	0.3226	0.0000	0.0000	0.0000	0.0000
Time	0.0000	0.0000	0.0000	0.0000	0.0000	0.0000
Enzyme × celeriac root	0.0000	0.0000	0.0000	0.0000	0.0000	0.0000
Enzyme × time	0.0000	0.1007	0.0000	0.0000	0.0001	0.0000
Seleriac root × time	0.0000	0.0001	0.0000	0.0000	0.0006	0.1216
Enzyme × celeriac root × time	0.0000	0.0493	0.0001	0.0000	0.0000	0.0000

Each value is expressed as mean ± SD (*n* = 3). Means in the same column with no common superscript differed significantly (*p* < 0.01) Abbreviation: P-juice pressed from peeled celery root, no enzyme, zero incubation time, without incubation in the incubator P1-juice pressed from peeled celery root, no enzyme, 30 min incubation in an incubator, incubation temperature of 25 °C; P2-juice pressed from peeled celery root, no enzyme, 60 min incubation in an incubator, incubation temperature of 25 °C; AP1-juice pressed from peeled celery root, enzyme α-amylase (dose: 15 mg/100 g pulp), 30 min incubation in an incubator, incubation temperature of 25 °C; AP2-juice pressed from peeled celery root, enzyme α-amylase (dose: 15 mg/100 g pulp), 60 min incubation in an incubator, incubation temperature of 25 °C; U-juice pressed from unpeeled celery root, no enzyme, zero incubation time, without incubation in the incubator; U1-juice pressed from unpeeled celery root, no enzyme, 30 min incubation in an incubator, incubation temperature of 25 °C; U2-juice pressed from unpeeled celery root, no enzyme, 60 min incubation in an incubator, incubation temperature of 25 °C; AU1-juice pressed from unpeeled celery root, enzyme α-amylase (dose: 15 mg/100 g pulp), 30 min incubation in an incubator, incubation temperature of 25 °C; AU2-juice pressed from unpeeled celery root, enzyme α-amylase (dose: 15 mg/100 g pulp), 60 min incubation in an incubator, incubation temperature of 25 °C.

**Table 2:** Content of certain flavonoids in celery juices.

Sample Name	Apigenin-6-*C*-Glucoside [mg/100 mL]	Kaempferol-3,7-Di-O-Glucoside [mg/100 mL]	Apigenin [mg/100 mL]	Chrysoeriol [mg/100 mL]	Chrysoeriol-7-*O*-Apiosylglucoside [mg/100 mL]	Total Flavonoids [mg/100 mL]
P	2.19 ^d^ ± 0.15	0.35 ^b^ ± 0.08	1.34 ^b^ ± 0.14	1.02 ^ab^ ± 0.02	0.61 ^d^ ± 0.07	5.50 ^a^ ± 0.10
P1	1.77 ^c^ ± 0.10	0.46 ^c^ ± 0.04	2.20 ^e^ ± 0.17	1.16 ^bc^ ± 0.07	0.12 ^a^ ± 0.02	5.72 ^a^ ± 0.20
P2	1.26 ^b^ ± 0.03	0.62 ^cd^ ± 0.09	2.20 ^e^ ± 0.09	1.36 ^c^ ± 0.07	0.20 ^ab^ ± 0.01	5.64 ^a^ ± 0.20
AP1	1.66 ^c^ ± 0.01	0.74 ^de^ ± 0.03	1.50 ^c^ ± 0.02	0.93 ^a^ ± 0.09	2.07 ^f^ ± 0.02	6.90 ^b^ ± 0.30
AP2	1.89 ^c^ ± 0.02	0.88 ^e^ ± 0.11	1.88 ^de^ ± 0.03	1.32 ^c^ ± 0.08	1.25 ^e^ ± 0.03	7.22 ^b^ ± 0.30
U	2.23 ^d^ ± 0.11	0.25 ^a^ ± 0.01	0.98 ^a^ ± 0.10	0.95 ^a^ ± 0.09	0.89 ^de^ ± 0.09	5.30 ^a^ ± 0.10
U1	1.81 ^c^ ± 0.14	0.40 ^bc^ ± 0.02	2.25 ^e^ ± 0.04	1.96 ^d^ ± 0.09	0.40 ^c^ ± 0.09	6.82 ^b^ ± 0.20
U2	0.85 ^a^ ± 0.17	0.57 ^cd^ ± 0.10	2.62 ^f^ ± 0.09	2.77 ^f^ ± 0.06	0.28 ^ab^ ± 0.03	7.07 ^b^ ± 0.10
AU1	2.57 ^e^ ± 0.10	0.65 ^cde^ ± 0.08	2.11 ^e^ ± 0.10	2.39 ^e^ ± 0.07	0.73 ^d^ ± 0.04	8.45 ^c^ ± 0.00
AU2	2.68 ^e^ ± 0.11	0.77 ^de^ ± 0.12	2.84 ^f^ ± 0.11	2.57 ^ef^ ± 0.04	0.20 ^ab^ ± 0.01	9.05 ^c^ ± 0.10
Three-factors analysis of variance ANOVA *p* <0.01						
Celeriac root	0.0000	0.0707	0.0000	0.0000	0.0000	0.0000
Enzyme	0.0000	0.0000	0.0000	0.7465	0.0000	0.0000
Time	0.0000	0.0029	0.0000	0.0000	0.0000	0.0619
Enzyme × celeriac root	0.0029	0.5440	0.0000	0.0073	0.0000	0.1385
Enzyme × time	0.0000	0.7281	0.0003	0.0140	0.0000	0.1842
Seleriac root × time	0.0000	0.9313	0.0005	0.0229	0.5908	0.2723
Enzyme × celeriac root × time	0.0643	0.8566	0.8564	0.0001	0.0050	0.9284

Each value is expressed as mean ± SD (*n* = 3). Means in the same column with no common superscript differed significantly (*p* < 0.01).

**Table 3:** Antioxidant activity and polyphenol content of celeriac juices.

Sample Name	ABTS˙^+^ [µmol TE/100 mL]	DPPH˙ [µmol TE/100 mL]	Total Polyphenol [mg GAE/100 mL]
P	483 ^c^ ± 9	68 ^e^ ± 1	15 ^b^ ± 1
P1	451 ^c^ ± 8	54 ^cd^ ± 3	13 ^a^ ± 1
P2	414 ^b^ ± 9	66 ^e^ ± 2	12 ^a^ ± 1
AP1	510 ^d^ ± 10	74 ^f^ ± 1	13 ^a^ ± 0
AP2	406 ^b^ ± 9	66 ^e^ ± 3	13 ^a^ ± 0
U	464 ^c^ ± 12	52 ^cd^ ± 4	22 ^c^ ± 1
U1	404 ^b^ ± 9	52 ^cd^ ± 4	18 ^b^ ± 1
U2	394 ^b^ ± 15	49 ^b^ ± 2	16 ^b^ ± 0
AU1	409 ^b^ ± 5	58 ^d^ ± 3	20 ^c^ ± 1
AU2	353 ^a^ ± 13	37 ^a^ ± 3	17 ^b^ ± 0
Three-factor analysis of variance ANOVA *p* < 0.01			
Celeriac root	0.0000	0.0000	0.0000
Enzyme	0.3943	0.0039	0.0056
Time	0.0000	0.0003	0.0000
Enzyme ×celeriac root	0.0001	0.0000	0.2790
Enzyme × time	0.0000	0.0000	0.1585
Celeriac root × time	0.0004	0.0000	0.0344
Enzyme × celeriac root × time	0.2232	0.9494	0.0805

Each value is expressed as mean ± SD (*n* = 3). Means in the same column with no common superscript differed significantly (*p* < 0.01).

**Table 4:** Press yield, extract, and carbohydrate content of celeriac juices.

Sample Name	Press Yield [%]	Extract [°Bx]	Total Sugars [g/100 mL]
P	47.3 ^a^ ± 0.5	6.90 ^ab^ ± 0.07	3.97 ^b^ ± 0.06
P1	64.0 ^ef^ ± 0.6	6.73 ^a^ ± 0.04	4.05 ^b^ ± 0.08
P2	66.7 ^f^ ± 2.0	6.87 ^ab^ ± 0.10	4.29 ^c^ ± 0.01
AP1	53.3 ^b^ ± 1.0	7.83 ^de^ ± 0.06	4.37 ^cd^ ± 0.10
AP2	72.3 ^g^ ± 0.9	7.57 ^cd^ ± 0.35	3.20 ^a^ ± 0.08
U	48.7 ^a^ ± 0.6	8.27 ^ef^ ± 0.10	4.66 ^e^ ± 0.04
U1	56.7 ^c^ ± 0.7	8.03 ^e^ ± 0.12	5.16 ^f^ ± 0.04
U2	60.0 ^d^ ± 1.0	8.10 ^e^ ± 0.10	4.56 ^e^ ± 0.02
AU1	62.7 ^de^ ± 0.8	7.33 ^c^ ± 0.15	4.30 ^c^ ± 0.00
AU2	70.7 ^g^ ± 0.2	7.20 ^bc^ ± 0.01	4.50 ^de^ ± 0.04
Three-factor analysis of variance ANOVA			
Celeriac root	0.0044	0.0000	0.0000
Enzyme	0.0000	0.4262	0.0000
Time	0.0000	0.4262	0.0000
Enzyme × celeriac root	0.0001	0.0000	0.1547
Enzyme × time	0.0000	0.0262	0.0000
Celeriac root × time	0.0000	0.7890	0.0000
Enzyme × celeriac root × time	0.0000	0.4262	0.0000

Each value is expressed as mean ± SD (*n* = 3). Means in the same column with no common superscript differed significantly (*p* < 0.01); total sugars: total fructose, glucose, and sucrose content.

## Data Availability

Data are contained within the article.

## Abbreviations

P	juice pressed from peeled celery root, no enzyme, zero incubation time, without incubation in the incubator.
P1	juice pressed from peeled celery root, no enzyme, 30 min incubation in an incubator, incubation temperature of 25 °C.
P2	juice pressed from peeled celery root, no enzyme, 60 min incubation in an incubator, incubation temperature of 25 °C.
AP1	juice pressed from peeled celery root, enzyme α-amylase (dose: 15 mg/100 g pulp), 30 min incubation in an incubator, incubation temperature of 25 °C.
AP2	juice pressed from peeled celery root, enzyme α-amylase (dose: 15 mg/100 g pulp), 60 min incubation in an incubator, incubation temperature of 25 °C.
U	juice pressed from unpeeled celery root, no enzyme, zero incubation time, without incubation in the incubator.
U1	juice pressed from unpeeled celery root, no enzyme, 30 min incubation in an incubator, incubation temperature of 25 °C.
U2	juice pressed from unpeeled celery root, no enzyme, 60 min incubation in an incubator, incubation temperature of 25 °C.
AU1	juice pressed from unpeeled celery root, enzyme α-amylase (dose: 15 mg/100 g pulp), 30 min incubation in an incubator, incubation temperature of 25 °C.
AU2	juice pressed from unpeeled celery root, enzyme α-amylase (dose: 15 mg/100 g pulp), 60 min incubation in an incubator, incubation temperature of 25 °C.
